# Eco-Friendly Bioinspired Synthesis and Environmental Applications of Zinc Oxide Nanoparticles Mediated by Natural Polysaccharide Gums: A Sustainable Approach to Nanomaterials Fabrication

**DOI:** 10.3390/nano16070407

**Published:** 2026-03-27

**Authors:** Jose M. Calderon Moreno, Mariana Chelu, Monica Popa

**Affiliations:** Institute of Physical Chemistry—Ilie Murgulescu, Romanian Academy, 202 Spl. Independentei, 060021 Bucharest, Romania; calderon@icf.ro

**Keywords:** natural gums, biopolymer-mediated synthesis, sustainable nanotechnology, polysaccharide stabilization, green nanomaterials, environmental remediation, nanoparticle toxicity, surface functionalization, gum arabic, ZnO

## Abstract

The green synthesis of nanomaterials has emerged as a sustainable and environmentally friendly approach, gaining significant attention in recent years for its potential in a wide range of multifunctional applications. Among these materials, zinc oxide nanoparticles (ZnO NPs) stand out due to their remarkable versatility and effectiveness in fields such as industry (food, chemistry, and cosmetics), nanomedicine, cancer therapy, drug delivery, optoelectronics, sensors, and environmental remediation. This study focuses on bioinspired strategies for the facile synthesis of ZnO NPs, employing natural polysaccharide gums as mediators. Acting as both reducing and stabilizing agents, natural gums not only facilitate the eco-friendly production of ZnO NPs but also enhance their stability and functionality. Natural gum-mediated green synthesis typically yields stable, spherical ZnO particles, often in the 10–100 nm range. Typical reaction conditions are the use of zinc acetate dihydrate or zinc nitrate (0.01–0.5 M) as precursors, with low gum concentrations of 0.1–1.0% (*w*/*v*) in distilled water, alkaline conditions (pH from 8 to 12), often achieved by adding NaOH, which aids in the reduction and capping by the gum, at reaction temperature between 60 °C and 80 °C, under continuous stirring. The dried precipitate is often calcined at 400 °C to 600 °C to remove organic residues and enhance crystallinity. This approach underscores the potential of biopolymer-assisted synthesis in advancing green nanotechnology for sustainable and practical applications. Utilizing environmentally benign materials such as natural gums for the synthesis of ZnO NPs offers significant advantages, including enhanced eco-friendliness and biocompatibility, making them suitable for a wide range of applications without the involvement of toxic reagents. This review provides an in-depth analysis of the synthesis and characterization techniques employed in the eco-friendly production of ZnO NPs using different natural gums from biological sources and its environmental applications (e.g., pollutant removal and increased agriculture sustainability).

## 1. Introduction

**Figure 1:**
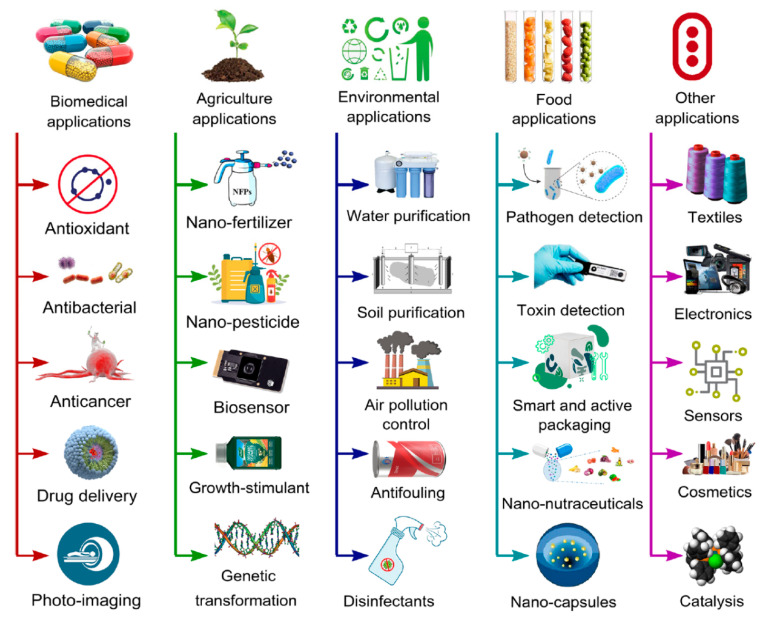
Potential applications of green ZnO NPs (reproduced from [6]).

Zinc oxide nanoparticles (ZnO NPs) have emerged as one of the most extensively investigated metal oxide nanomaterials owing to their unique physicochemical properties, multifunctionality, and relative abundance [1,2]. ZnO is a wide bandgap semiconductor with high exciton binding energy, excellent chemical stability, and strong antimicrobial, antioxidant, and photocatalytic activity. These attributes have enabled its widespread application across diverse fields (Figure 1), including biomedicine, agriculture, water and soil purification for environmental remediation, food packaging, cosmetics, nanonutraceuticals, optoelectronics, sensors, smart wearables, and photocatalysis [3,4,5,6].

At the nanoscale, ZnO exhibits size- and morphology-dependent properties that significantly outperform its bulk counterpart, further accelerating research interest [7]. Despite these advantages, the rapid expansion of ZnO nanoparticle production has raised critical concerns regarding sustainability, environmental safety, and human health. Conventional synthesis techniques—such as sol–gel processing, hydrothermal synthesis, chemical vapor deposition, and precipitation methods—often rely on toxic solvents, hazardous reducing agents, high energy consumption, and stringent reaction conditions [8]. These approaches contradict the principles of green chemistry and pose challenges related to waste management, environmental contamination, and potential cytotoxicity of residual chemicals. Consequently, the development of environmentally benign and sustainable synthesis strategies has become an urgent priority in nanotechnology research [9].

**Figure 2:**
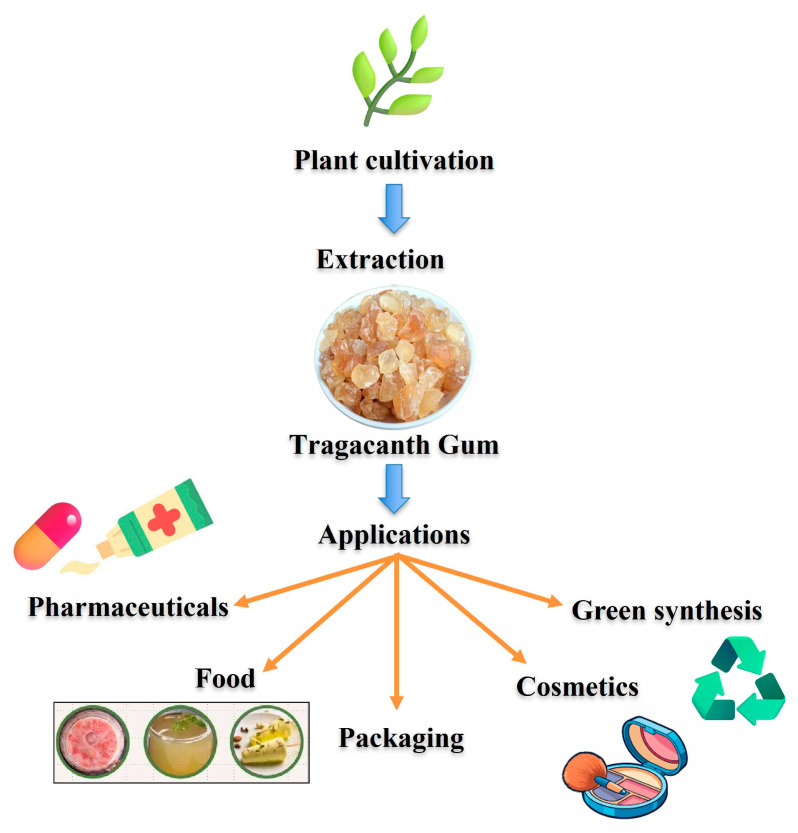
Plant-based tragacanth gum as sustainable biopolymer (reproduced from [16]).

Green synthesis has emerged as a promising alternative, emphasizing the use of renewable, non-toxic, and biodegradable resources for nanoparticle fabrication [10]. Biological materials such as plant extracts, polysaccharides, proteins, microorganisms, and biopolymers have been explored as reducing, capping, and stabilizing agents [6,8,11,12,13]. Among these, natural gums—plant-derived polysaccharides obtained from exudates, seeds, or microbial fermentation—have attracted increasing attention due to their chemical versatility, abundance, low cost, and excellent biocompatibility (Figure 2) [14,15,16]. Natural gums possess a high density of functional groups, including hydroxyl, carboxyl, and acetyl moieties, which facilitate metal ion chelation [17].

Natural gum-mediated synthesis of ZnO NPs represents a particularly attractive strategy because it integrates reduction, stabilization, and surface functionalization into a single step while operating under mild reaction conditions [18,19]. The resulting nanoparticles often exhibit improved dispersion stability, controlled size distribution, and enhanced biological compatibility [20]. Moreover, the use of natural gums aligns well with circular economy principles by valorizing renewable resources and reducing chemical waste.

This review critically examines the eco-friendly synthesis of ZnO NPs mediated by natural gums. The first part of the article introduces the fundamental properties and technological significance of ZnO NPs, followed by an evaluation of the limitations associated with conventional synthesis methods. Subsequent sections focus on the chemistry of natural gums, their mechanistic role in nanoparticle formation, process parameters governing particle characteristics, comparative assessment with other green synthesis routes, and environmental applications. Finally, current challenges, knowledge gaps, and future research directions are briefly discussed to provide an outlook on this emerging and sustainable nanotechnology platform.

## 2. ZnO NPs: Structure, Properties, and Functional Significance

Zinc oxide is an n-type semiconductor that crystallizes predominantly in the hexagonal wurtzite structure, which is thermodynamically stable under ambient conditions. At the nanoscale, ZnO typically exhibits particle sizes ranging from a few nanometers to several tens of nanometers and can adopt diverse morphologies, including spheres, rods, wires, plates, flowers, and hierarchical architectures [21]. This structural versatility is closely linked to synthesis conditions and has a profound influence on the material’s optical, electronic, and surface properties [22].

One of the most significant characteristics of ZnO NPs is their wide direct band gap (~3.37 eV at room temperature) and high exciton binding energy (~60 meV) [2,23]. These features enable strong ultraviolet absorption and emission, making ZnO NPs highly attractive for optoelectronic devices, UV photodetectors, and light-emitting diodes. Quantum confinement effects at reduced particle sizes further allow tunability of the band gap and photoluminescence behavior, which is critical for sensor and imaging applications [24].

**Figure 3:**
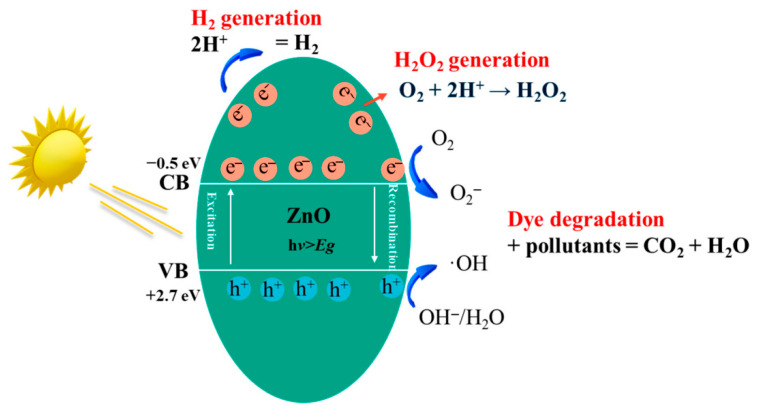
Schematic photocatalytic mechanism for ZnO NPs (reproduced from [29]).

ZnO NPs also exhibit remarkable surface reactivity due to their high surface-to-volume ratio. Surface defects, oxygen vacancies, and zinc interstitials play a key role in governing photocatalytic and antimicrobial activities [25]. Under UV or visible light irradiation, ZnO NPs generate electron–hole pairs that participate in redox reactions, producing reactive oxygen species, including H_2_ an H_2_O_2_, capable of degrading organic pollutants (Figure 3). This property has been widely exploited in wastewater treatment and environmental remediation [26,27,28,29].

**Figure 4:**
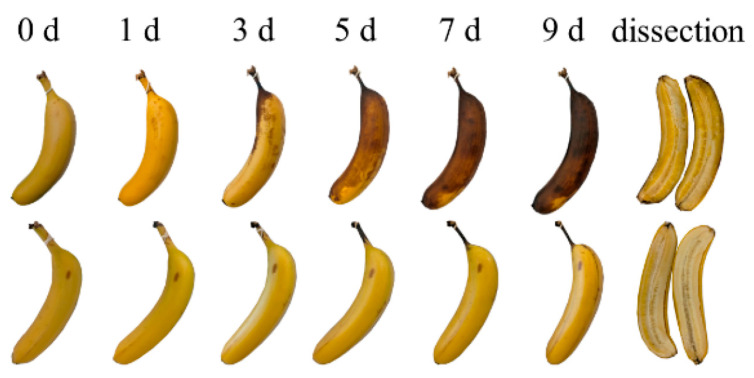
Changes in the visual appearance of bananas during storage (reproduced from [31]).

From a biological perspective, ZnO NPs demonstrate broad-spectrum antimicrobial activity against bacteria, fungi, and some viruses [30]. The proposed mechanisms include membrane disruption, oxidative stress induction, and the release of Zn^2+^ ions [4,21]. These features have led to their incorporation into wound dressings, coatings, food packaging, and personal care products. Figure 4 shows the effectiveness of a locust bean gum/ZnO coating applied to bananas for preservation. As depicted in the upper row of images in Figure 4, the uncoated banana darkens and turns brown after 9 days of storage due to enzymatic production of melanin. Consequently, the banana becomes unsuitable for consumption. Coated bananas, shown in the lower row of images in Figure 4, exhibit darkening of the stems, while the skin and the flesh remain fresh due to the excellent oxygen barrier and antioxidant properties of the coating, which efficiently reduces the enzymatic browning reaction [31].

Importantly, ZnO is generally recognized as safe at controlled concentrations, further supporting its biomedical relevance [32]. Green ZnO NPs biomedical applications are well-documented in the recent literature [33,34]. The functional significance of ZnO NPs is also evident in agriculture, where they are explored as nanofertilizers, growth promoters, and antimicrobial agents for plant protection [35]. In energy-related applications, ZnO NPs serve as photoanodes in dye-sensitized solar cells and components in piezoelectric and triboelectric nanogenerators [36,37].

However, the performance of ZnO NPs is highly sensitive to particle size, morphology, surface chemistry, and defect density. These parameters are, in turn, dictated by the synthesis route employed [22,38]. Therefore, the choice of synthesis strategy is not merely a preparative step but a decisive factor determining the applicability, safety, and sustainability of ZnO nanomaterials.

## 3. Limitations of Conventional ZnO Nanoparticle Synthesis Methods

Conventional synthesis methods for ZnO NPs have been extensively developed and optimized to achieve precise control over particle size, morphology, and crystallinity. Common techniques include sol–gel processing, hydrothermal and solvothermal synthesis, chemical precipitation, thermal decomposition, and vapor phase deposition methods [5]. While these approaches are effective in producing high-purity ZnO NPs, they suffer from several critical limitations that restrict their sustainability and large-scale applicability. One of the primary drawbacks of conventional methods is the reliance on toxic chemicals and hazardous solvents. Conventional ZnO nanoparticle synthesis is associated with poor environmental compatibility [39]. Strong reducing agents, organic solvents, surfactants, and stabilizers are frequently employed to control nucleation and growth [40]. Residual chemicals adsorbed on nanoparticle surfaces can pose serious environmental and health risks, particularly in biomedical and environmental applications. Effluents generated during chemical synthesis may contain unreacted precursors, heavy metal ions, and toxic byproducts that require careful disposal. Inadequate waste management can result in soil and water contamination, undermining the environmental benefits of ZnO-based technologies. The removal of these residues often requires extensive purification steps, increasing water consumption and waste generation [41]. Energy-intensive processing is another major concern. Many conventional synthesis routes require high temperatures, elevated pressures, or prolonged reaction times, leading to substantial energy consumption and increased carbon footprint. For instance, hydrothermal and solvothermal methods often operate above 150–200 °C, while vapor phase techniques demand sophisticated infrastructure and high operational costs. These factors hinder scalability and industrial adoption, especially in resource-limited settings [42]. From a functional standpoint, chemically synthesized ZnO NPs may exhibit limited biocompatibility due to surface-bound toxic ligands [43]. This restricts their direct use in biomedical and environmental applications without further surface modification. Additionally, some conventional methods offer limited control over agglomeration. Their high surface reactivity predisposes them to aggregation, leading to reduced surface area, which complicates their dispersion in aqueous and biological systems [44,45] and compromises performance due to instability under physiological conditions and concerns about cytotoxicity and environmental persistence [15] diminishing functional efficiency. Uncoated ZnO NPs can generate reactive oxygen species (ROS), leading to oxidative stress in living systems [46]. Therefore, strategies to modulate their surface chemistry and improve their biocompatibility are essential for safe and effective utilization.

These limitations have prompted a paradigm shift toward greener synthesis strategies that minimize chemical hazards, reduce energy input, and enhance biocompatibility. Natural gum-mediated synthesis represents one such promising alternative, offering a sustainable route to ZnO NPs while maintaining functional performance. By addressing the shortcomings of conventional approaches, green synthesis strategies aim to reconcile nanomaterial innovation with environmental responsibility and regulatory compliance.

The encapsulation of ZnO NPs within biocompatible carriers is a promising approach to mitigating toxicity and improving stability. Natural gums, plant- or microbial-derived polysaccharides such as gum arabic/acacia, and guar gum offer an eco-friendly and functional matrix for encapsulation. These biopolymers are biodegradable, non-toxic, and capable of forming gels or films that can entrap nanoparticles effectively [44,47]. Moreover, encapsulation can enhance dispersion, modulate release kinetics, and reduce nanoparticle interaction with non-target biological structures. In some cases, the gums themselves contribute additional antimicrobial or antioxidant properties, enhancing the overall functional performance of the nanocomposite [48,49]. Although a wide spectrum of biological resources—including plant extracts [50,51,52,53,54,55,56,57,58], algae [59], microorganisms [60,61], proteins [62,63], and fungi [64]—has been successfully employed in the green synthesis of ZnO NPs, the present review deliberately confines its scope to natural gums as sustainable mediators. This focus is justified on both mechanistic and practical grounds. Although both plant extracts and gums are chemically heterogeneous and often exhibit batch-to-batch variability due to differences in phytochemical composition, seasonal factors, and extraction procedures, natural gums are more structurally defined, high-molecular-weight polysaccharides with comparatively consistent composition and physicochemical behavior. Their polymeric architecture, rich in hydroxyl and carboxyl functional groups, enables predictable metal ion chelation, controlled nucleation, and steric stabilization—features that are particularly advantageous for reproducible nanoparticle synthesis and scalable processing. Moreover, gums simultaneously act as complexing agents, growth-directing matrices, and surface stabilizers, thereby integrating multiple functional roles into a single, renewable material platform. While plant extract-mediated synthesis may offer strong reducing capability and enhanced surface bioactivity, its intrinsic compositional variability can complicate mechanistic interpretation and industrial standardization. By concentrating exclusively on natural gums, this review aims to provide a coherent and mechanistically focused analysis of a subclass of green mediators that combine sustainability, structural uniformity, and process controllability. Such a targeted approach allows for deeper insight into structure–function relationships, reaction parameter optimization, and application-specific design strategies within gum-mediated ZnO nanoparticle systems rather than offering a broad overview of all biogenic synthesis routes.

## 4. Natural Gums as Sustainable Mediators: Sources, Chemical Composition, and Physicochemical Properties

### 4.1. Sources and Classification of Natural Gums

Natural gums are high-molecular-weight polysaccharides derived from various biological sources, including plant exudates (e.g., gum arabic and gum karaya), seeds (e.g., guar gum and locust bean gum), marine organisms (e.g., alginate and carrageenan) and microbial fermentation products (e.g., xanthan gum and gellan gum) [65]. Plant exudate gums, such as gum arabic, gum tragacanth, and gum karaya, are secreted by trees in response to injury [66]. Seed gums, including guar gum and locust bean gum, are extracted from endosperms of leguminous plants [67]. Microbial gums, such as xanthan and gellan gum, are produced through fermentation processes, while marine gums like alginate and carrageenan are derived from seaweeds [68]. Although cellulose and starch themselves are not natural gums, their chemical or enzymatic modification can produce water-soluble derivatives with gum-like properties, widely used in various industries. These cellulose derivatives (e.g., carboxymethyl cellulose and microcrystalline cellulose) or starch-derived gums, such as dextran, maltodextrin, and cyclodextrins, are widely used for their thickening, stabilizing, and encapsulating capabilities [69]. Chitosan, a natural polysaccharide with gelling and viscosifying properties similar to gums, is considered as a cationic polysaccharide biopolymer derived from chitin rather than a natural gum [70].

**Figure 5:**
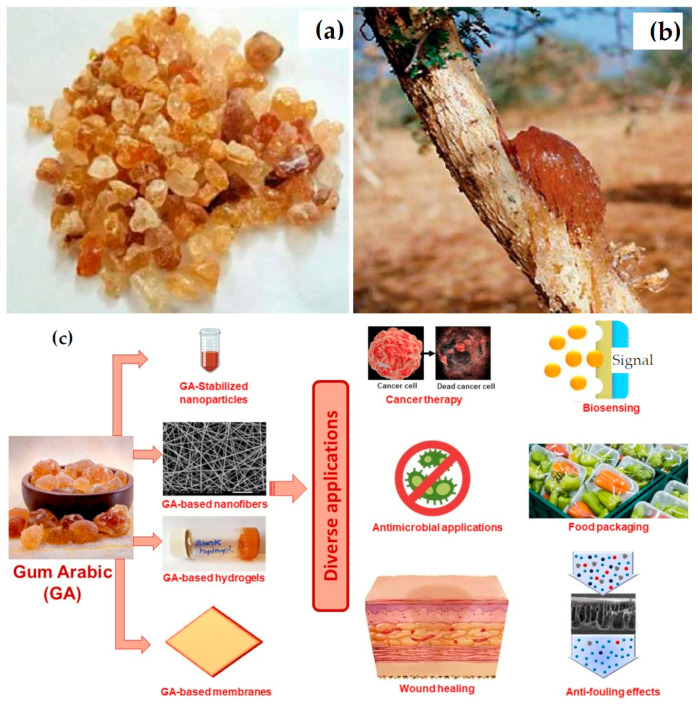
(**a**) Gum arabic exudates from (**b**) acacia tree secretions (reproduced from [76]); (**c**) gum arabic versatile formulations and applications (reproduced from [74]).

For centuries, natural gums they have been used in food, pharmaceutical, textile, and cosmetic industries as thickening, stabilizing, emulsifying, and binding agents [71,72]. In recent years, their intrinsic biocompatibility, renewability, and multifunctional chemical nature have positioned them as promising materials in green nanotechnology, particularly as mediators in nanoparticle synthesis [73,74]. Plant-based gums, such as gum arabic and guar gum, are well-known for their excellent emulsification and thickening properties. In contrast, microbial gums, such as xanthan and gellan, offer unique rheological behaviors that are beneficial for stabilizing colloidal systems. Marine-derived gums, including alginate and carrageenan, are widely used for their gel-forming capabilities, especially in biomedical applications. One of the most compelling reasons for employing natural gums in nanotechnology is their inherent biocompatibility and biodegradability. Figure 5 illustrates diverse gum arabic formulations and applications. Gums like gum arabic and guar gum have long histories of safe use in food and pharmaceutical formulations [74,75]. Their degradation products are non-toxic and can be metabolized or eliminated by biological systems without creating harmful residues. This makes them suitable for biomedical and environmental applications, particularly where long-term exposure or bioaccumulation is a concern. These characteristics can be tailored by blending different gums or modifying their chemical structure to optimize encapsulation efficiency and nanoparticle compatibility. Their structural heterogeneity and functional group diversity provide a versatile platform for interacting with metal ions and controlling nanoparticle growth.

**Figure 6:**
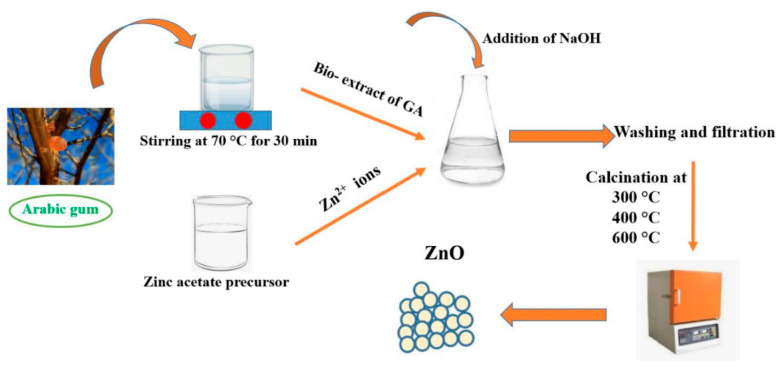
A scheme of gum arabic-mediated fabrication of ZnO NPs (reproduced from [28]).

Several plant-derived and microbial gums, particularly gum arabic, have been experimentally validated as effective mediators for green ZnO nanoparticle synthesis (Figure 6). Table 1 highlights recent scientific reports and identifies for the natural gum used, its primary source, major components, and role in ZnO nanoparticle synthesis for each one. Among these, plant and seed gums are most commonly explored due to their wide availability, low cost, and minimal processing requirements.

Figure 6 shows a scheme of gum arabic-mediated fabrication of ZnO NPs from [28]. Gum arabic and zinc acetate were separately dissolved in distilled water and subsequently mixed and kept under stirring for 10 min to obtain the equilibrium state. A total of 1 M NaOH was then added dropwise to the mixture, and the solution was kept under stirring for 2 h to obtain the white precipitate. Finally, the solution was filtered, washed, and dried at 70 °C for 6 h, followed by calcination at 300 °C, 400 °C, and 600 °C in air for 2 h to obtain a white powder of ZnO NPs.

**Figure 7:**
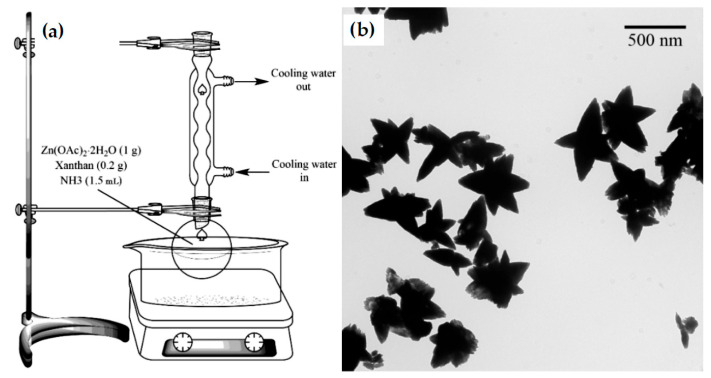
(**a**) A scheme of the natural gum-mediated synthesis and (**b**) TEM image of Xanthan gum-capped zinc oxide (ZnO) microstars (reproduced from [83]).

Figure 7 illustrates natural gum-mediated synthesis of ZnO using xanthan gum and Zn(OAc)_2_·2H_2_O (as Zn^2+^ source) dissolved in ultrapure water. NH_4_OH was added dropwise to the solution, and the solution was refluxed for 6 h at 80 °C, centrifuged, refluxed for 9 h, washed, and dried [83].

**Figure 8:**
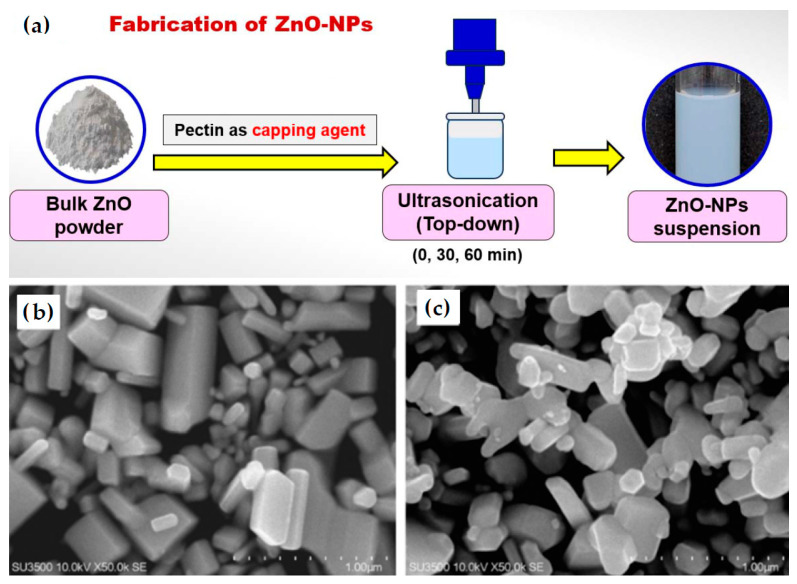
Pectin-capped ZnO NPs obtained through ultrasonication in double-distilled water: (**a**) a scheme of the natural gum-mediated fabrication method and SEM images of ZnO-NPs after ultrasonication for (**b**) 30 m and (**c**) 60 m (reproduced from [90]).

Astriyani et al. [90] used a top-down method starting from ZnO powder and, through ultrasonication, obtained ZnO-NPs using pectin as a capping agent (Figure 8a). ZnO powder and pectin were dissolved in double-distilled water and ultrasonicated using a probe-type sonicator (300 W, 20 kHz) at 70% amplitude. After 30 m of ultrasonication, ZnO NPs were well dispersed, exhibiting a rod-shaped morphology and showing defined crystalline edges (Figure 8b). After 60 m of ultrasonication (Figure 8c), the NPs showed reduced size and particle clustering.

### 4.2. Chemical Composition and Functional Groups

Natural gums are primarily composed of complex polysaccharide chains consisting of monosaccharide units such as arabinose, galactose, mannose, rhamnose, xylose, and glucose. These units are linked through glycosidic bonds to form branched or linear macromolecular architectures. In addition to carbohydrate backbones, many gums contain associated proteins, uronic acids, acetyl groups, and inorganic ions [74,76,106,107,108]. The abundance of hydroxyl (–OH), carboxyl (–COOH), carbonyl (–C=O), and occasionally sulfate (–SO_3_H) groups is particularly significant for nanoparticle synthesis [44]. These functional moieties enable strong coordination with Zn^2+^ ions, facilitate controlled hydrolysis, and stabilize growing ZnO nuclei through steric and electrostatic interactions. The presence of uronic acids and negatively charged groups enhances metal ion chelation, making gums effective complexing agents in aqueous systems.

An aspect that must be acknowledged is the intrinsic variability in the chemical composition of natural gums, which has direct implications for the reproducibility and standardization of gum-mediated ZnO nanoparticle synthesis. Natural gums such as gum arabic, guar gum, xanthan gum, and tragacanth are complex, heterogeneous polysaccharide systems composed of varying proportions of monosaccharides (e.g., arabinose, galactose, rhamnose, and mannose), uronic acids, proteins, and mineral impurities. Their exact composition depends strongly on botanical origin, geographical location, climatic conditions, harvesting season, and post-extraction processing methods. Even within the same nominal gum type (e.g., gum arabic), batch-to-batch variability can be significant due to differences in plant species (e.g., *Acacia senegal* vs. *Acacia seyal*) and purification levels. This compositional heterogeneity directly affects their function as reducing, capping, and stabilizing agents in ZnO nanoparticle synthesis. Variations in functional group density (–OH, –COOH), molecular weight distribution, and branching structure can influence the rate of nucleation and growth of ZnO nanoparticles, the extent of surface passivation and aggregation control, and the resulting particle morphology, crystallinity, and surface chemistry. Consequently, two studies employing the “same” gum may yield different nanoparticle characteristics and performance metrics, even under similar experimental conditions. This presents a fundamental limitation when interpreting and comparing results across the literature.

### 4.3. Physicochemical Properties Relevant to Nanoparticle Synthesis

Natural gums exhibit several advantageous properties that make them excellent encapsulating agents for nanoparticles. These polysaccharides provide multifunctional roles through metal ion chelation, steric stabilization, nucleation, and controlled nanoparticle growth [109]. They are widely recognized for their biocompatibility, biodegradability, and capacity to stabilize dispersed systems [110]. Their molecular structure often includes branching, which influences solubility and film-forming capacity—key traits relevant to nanoparticle encapsulation, including ZnO. Functional groups (hydroxyl, carboxyl, etc.) in the polymer chains can interact with metal oxides like ZnO, aiding in surface modification and stabilization. Their hydrophilicity enables easy dispersion in aqueous systems and supports the formation of colloidal suspensions, while their viscosity, gel formation ability, and adhesiveness are useful for surface coating of nanoparticles or forming biopolymer shells to provide structural integrity to encapsulated systems and help in sustained nanoparticle release applications.

The physicochemical behavior of natural gums in solution plays a critical role in governing nanoparticle formation. Key properties include solubility, viscosity, molecular weight, charge density, and thermal stability. Most natural gums readily dissolve or swell in water, forming viscous colloidal solutions that provide a confined reaction environment for nucleation and growth of nanoparticles. High molecular weight and branched structures impart steric hindrance, which helps prevent nanoparticle aggregation. Meanwhile, the polyelectrolyte nature of many gums contributes to electrostatic stabilization, resulting in well-dispersed ZnO NPs. Viscosity can be tuned by adjusting gum concentration, offering an additional handle to regulate particle size and morphology. Thermal stability of natural gums is sufficient for mild synthesis conditions (typically below 100 °C), aligning well with green synthesis protocols. Upon calcination or thermal treatment, organic components are decomposed, yielding crystalline ZnO while often preserving nanoscale features.

### 4.4. Sustainability and Advantages over Synthetic Polymers

From a sustainability perspective, natural gums offer several advantages over synthetic polymers traditionally used as capping agents. They are biodegradable, non-toxic, and derived from renewable resources. Their production generally requires less energy and generates fewer hazardous byproducts. In nanoparticle synthesis, natural gums act simultaneously as reducing agents, stabilizers, and surface modifiers, eliminating the need for multiple additives. This multifunctionality simplifies synthesis protocols, reduces chemical load, and enhances the environmental compatibility of the final nanomaterial. The use of natural gums reduces the dependence on petroleum-based chemicals and aligns with green chemistry principles [17]. These attributes make natural gums particularly attractive mediators for eco-friendly ZnO nanoparticle synthesis.

## 5. Mechanism of Natural Gum-Mediated ZnO Nanoparticle Synthesis

The synthesis of ZnO NPs mediated by natural gums involves a combination of physicochemical processes driven by metal–biopolymer interactions, hydrolysis, nucleation, and controlled growth. Unlike conventional chemical routes, gum-mediated synthesis integrates reduction, stabilization, and surface functionalization into a unified framework operating under mild conditions [18,33]. The initial step in gum-mediated ZnO nanoparticle synthesis involves the interaction between Zn^2+^ ions and functional groups present in the gum matrix. Carboxylate, hydroxyl, and carbonyl groups act as ligands, providing sites for the binding of zinc ions through electrostatic interactions and chelation, forming stable metal–polymer complexes. This complexation reduces the free ion concentration in solution, thereby modulating the rate of hydrolysis and preventing uncontrolled precipitation. which would otherwise lead to large, irregular particles. The formation of these complexes serves as a precursor activation stage, ensuring homogeneous distribution of zinc ions throughout the biopolymer network. This spatial confinement is critical for achieving uniform nucleation and narrow particle size distributions [19,111].

Upon pH adjustment or thermal treatment, zinc–gum complexes undergo hydrolysis to form zinc hydroxide intermediates. Subsequent dehydration leads to the formation of ZnO nuclei. The nucleation process is strongly influenced by the density and accessibility of functional groups within the gum structure [112,113]. Natural gums adsorb onto the surface of newly formed ZnO nuclei, acting as capping agents that inhibit excessive growth and aggregation [101]. Steric hindrance provided by the bulky polysaccharide chains limits particle coalescence, while electrostatic repulsion between charged groups further stabilizes the colloidal system. This dual stabilization mechanism enables controlled growth and shape evolution of ZnO NPs.

**Figure 9:**
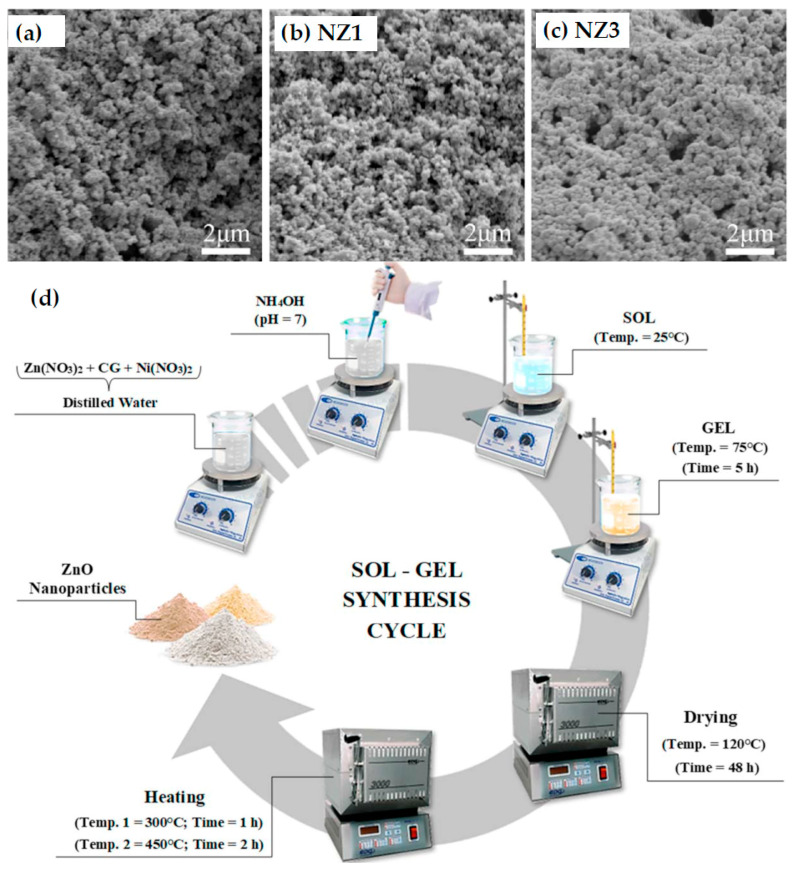
SEM images of (**a**) undoped ZnO NPs and Ni-doped Zn_1−x_Ni_x_O NPs, (**b**) NZ1 (x = 0.01), (**c**) NZ3 (x = 0.03), synthesized using cashew gum, and (**d**) a scheme of the natural gum-mediated synthesis (reproduced from [102]).

Several synthetic strategies have been employed for gum-mediated ZnO nanoparticle fabrication, including precipitation [12,14,18,28,77,78,83,84,86,87,88,96,97,103,104,113,114,115,116], microwave-assisted methods [11,112,117], and sol–gel-like methods [44,91,102,118], under green conditions and top-down approaches [90]. In a typical approach, an aqueous solution of zinc salt is mixed with a gum solution, followed by controlled pH adjustment using mild bases. The reaction is conducted at relatively low temperatures, often below 90 °C. Figure 9 highlights the natural gum-mediated synthesis using cashew gum, Zn(NO_3_)_2_ and Ni(NO_3_)_2_, disolved in distilled water. By adding NH_4_OH dropwise, the pH was adjusted to 7 and the solutions were heated to 75 °C for 5 h under constant stirring. The formed gels were dried for 48 h at 120 °C and then calcined [102].

Not all natural gums perform equally as stabilizing agents. Stabilization efficiency depends on functional group density, molecular architecture, and solubility. Gum arabic is widely regarded as one of the most effective stabilizers due to its highly branched arabinogalactan structure and the presence of uronic acids (–COO^−^ groups). These features provide both electrostatic and steric stabilization, leading to well-dispersed ZnO NPs with minimal aggregation. Xanthan gum also exhibits strong stabilization due to its rigid backbone and charged side chains, which promote extended conformations in solution and effective surface coverage of nanoparticles. Guar gum, primarily composed of galactomannan, provides good steric stabilization but lacks significant charge density, making it less effective in preventing aggregation under high ionic strength conditions. Tragacanth gum, rich in acidic polysaccharides, can provide strong binding but often leads to higher viscosity systems, which may hinder uniform nucleation. Overall, gums containing both hydroxyl and carboxyl functional groups with moderate molecular weight and branching (e.g., gum arabic) tend to offer the best balance between stabilization efficiency and processability.

The nucleation of ZnO NPs in gum-mediated systems is strongly influenced by coordination interactions between Zn^2+^ ions and polysaccharide functional groups. Hydroxyl groups (–OH) act as weak ligands, forming hydrogen-bonded or coordination interactions with Zn^2^. Carboxyl groups (–COO^−^) provide stronger coordination, forming Zn–carboxylate complexes that act as localized nucleation centers. This coordination has two major effects: binding of Zn^2+^ ions delays their free hydrolysis, reducing the rate of homogeneous nucleation, while localized Zn^2+^–polymer complexes promote spatially controlled nucleation along the polymer chain (heterogeneous nucleation sites). As a result, gums with higher carboxylate content typically produce more uniform and smaller nanoparticles, as nucleation is better regulated and spatially distributed.

Steric stabilization arises from the adsorption of polymer chains onto nanoparticle surfaces, creating a physical barrier that prevents particle–particle contact. In gum-mediated systems, polysaccharide chains adsorb onto ZnO nuclei via coordination or hydrogen bonding. The hydrated polymer layer extends into the surrounding medium, generating entropic repulsion when particles approach each other. This prevents van der Waals-driven aggregation and limits particle growth. Highly branched gums (e.g., gum arabic) are particularly effective because they form thick, multi-point attachment layers, increasing steric hindrance. In contrast, linear polymers may provide thinner coverage and less effective stabilization.

Electrostatic stabilization is governed by the surface charge imparted by ionizable functional groups, primarily carboxylates. At alkaline pH, –COOH groups deprotonate to –COO^−^, imparting negative charge to the nanoparticle surface. This generates electrostatic repulsion between particles, reducing aggregation. The effectiveness of this mechanism depends on charge density of the gum and ionic strength, which can screen electrostatic interactions. Gums such as gum arabic and xanthan gum, which contain significant acidic residues, provide stronger electrostatic stabilization compared with neutral gums like guar gum. However, electrostatic stabilization alone is often insufficient under environmental conditions; thus, combined electrosteric stabilization (electrostatic + steric) is typically the dominant mechanism.

Viscosity, governed by gum concentration and molecular weight, plays a crucial but often overlooked role in nanoparticle formation. Increased viscosity reduces diffusion rates of Zn^2+^ and OH^−^ ions, slowing nucleation and growth kinetics. This can lead to more controlled and homogeneous nucleation, producing narrower size distributions. However, excessively high viscosity can limit mixing efficiency, create localized supersaturation zones, and result in heterogeneous particle size distribution. Thus, viscosity introduces a trade-off between kinetic control and mass transfer limitations. Gums such as guar and tragacanth, which significantly increase solution viscosity even at low concentrations, require careful optimization to avoid non-uniform particle formation. In contrast, gum arabic provides moderate viscosity with effective stabilization, contributing to its frequent selection in ZnO synthesis studies.

In some cases, post-synthesis thermal treatment or calcination is applied to remove the organic matrix and enhance crystallinity [28]. Careful optimization of calcination temperature is necessary to balance organic removal with preservation of nanoscale features.

**Figure 10:**
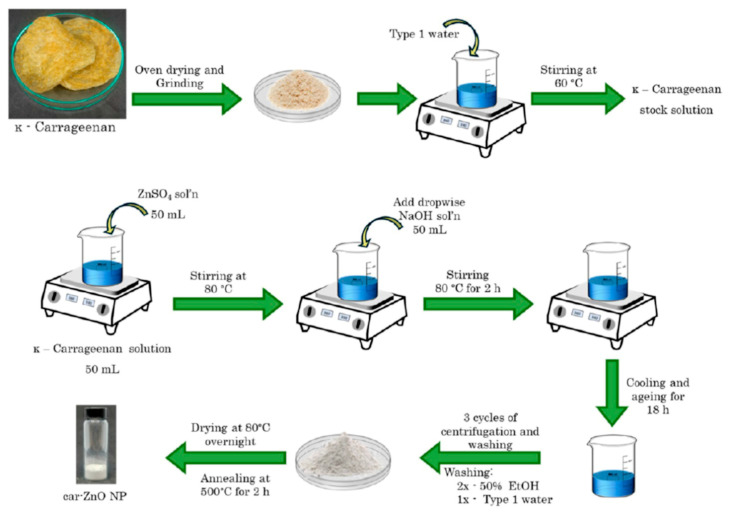
A schematic of the *κ*-carrageenan-mediated synthesis of ZnO NPs (reproduced from [86]).

Figure 10 is a schematic of the synthesis of ZnO NPs using *κ*-carrageenan, carried out by mixing 50 mL of 0.25 M zinc sulfate heptahydrate solution with varying concentrations of 50 mL of aqueous carrageenan solution (0.00% *w*/*v*, 0.25% *w*/*v*, 0.50% *w*/*v*, 0.75% *w*/*v*, and 1.00% *w*/*v*). NaOH solution (0.5 M) was added dropwise, while the solution was vigorously mixed at 80 °C for 2 h. The mixture was then aged for 18 h. After being aged, the precipitate was centrifuged and subsequently washed with water and ethanol. The washed samples were dried in an oven at 80 °C overnight and annealed in a muffle furnace for 2 h at 500 °C [86].

The mechanistic sequence underlying natural gum-mediated ZnO NPs formation is presented stepwise in the following schematic reaction pathway:

Step 1: The dissolution of Zn^2+^ ions. A zinc precursor (commonly zinc nitrate, zinc acetate, zinc chloride, or zinc sulfate) is dissolved in an aqueous medium, generating hydrated Zn^2+^ ions.

Step 2: The coordination of Zn^2+^ with polysaccharide chains. Simultaneously, natural gum polysaccharides disperse in solution, forming a hydrated polysaccharide network rich in functional groups—hydroxyl (–OH) and carboxylate (–COO^−^) groups. These functional groups on the gum backbone coordinate Zn^2+^ ions through ligand exchange. This coordination forms polymer–metal complexes that act as localized reservoirs of zinc ions and serve as heterogeneous nucleation sites.

Step 3: Controlled hydrolysis and the formation of zinc hydroxide intermediates. Under alkaline conditions, upon the addition of a base (e.g., NaOH or NH_4_OH), hydroxide ions induce hydrolysis of coordinated Zn^2+^. In the absence of gums, this step would lead to rapid precipitation and aggregation. However, polymer coordination slows the hydrolysis kinetics, promoting controlled nucleation. The gum matrix controls supersaturation and nucleation rate.

Step 4. The nucleation of ZnO nanocrystals. Upon heating or aging, zinc hydroxide undergoes dehydration and structural rearrangement to form ZnO nuclei. This process may occur during thermal treatment or aging at elevated temperature. The gum matrix confines nucleation events along the polymer chain, producing spatially distributed, nanoscale ZnO nuclei.

Step 5: Growth and stabilization/surface passivation. As nucleation proceeds, ZnO nanocrystals grow through the diffusion of additional Zn^2+^ and OH^−^ species. Simultaneously, polysaccharide chains adsorb onto the nanoparticle surface. This adsorption forms a hydrated polymer shell that blocks further uncontrolled growth, prevents particle–particle contact, and stabilizes nanoparticles through steric stabilization, creating a hydrated steric barrier that prevents aggregation and electrostatic stabilization, whereas ionized carboxyl groups provide surface charge and electrostatic repulsion.

Step 6: Drying or calcination (optional). Depending on synthesis conditions, the gum may remain as a surface coating or be partially decomposed during calcination. The calcination improves crystallinity and photocatalytic activity but reduces organic surface functionality.

## 6. Effect of Reaction Parameters on Nanoparticle Characteristics and Physicochemical Properties

**Figure 11:**
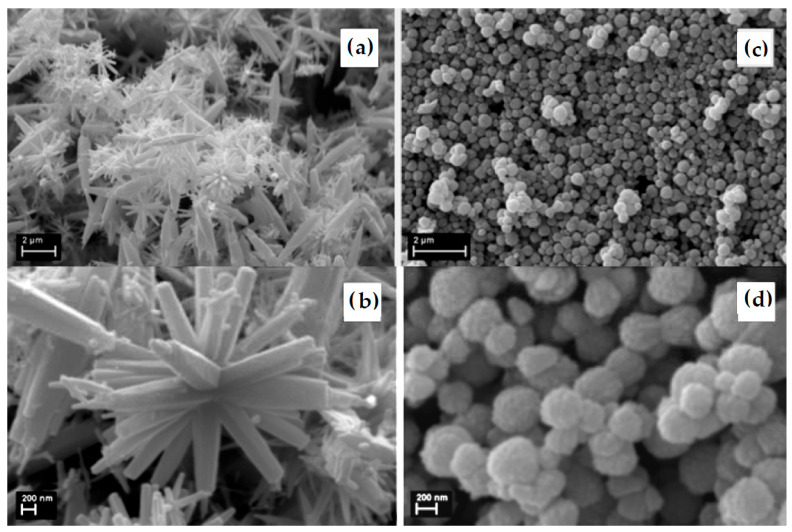
SEM images of ZnO crystallites prepared from Zn(NO_3_)_2_ (**a**,**b**) in the absence of gum arabic and (**c**,**d**) in the presence of gum arabic (reproduced from [116]).

The physicochemical characteristics of gum-mediated ZnO NPs are highly sensitive to synthesis parameters. Fine-tuning these variables is essential to achieve the desired particle size, morphology, crystallinity, and functional performance. The mechanistic versatility of natural gums enables precise control over ZnO nanoparticle formation while maintaining eco-friendly synthesis conditions. Beyond synthesis, natural gums impart surface functionality to ZnO NPs. Residual polysaccharide layers or functional groups on the nanoparticle surface can enhance dispersion stability, biocompatibility, and interaction with biological or environmental targets. This intrinsic surface modification is particularly advantageous for applications in antimicrobial coatings, drug delivery, and environmental remediation [81]. Figure 11 illustrates the influence of gum arabic on the shape of ZnO crystallites prepared from Zn(NO_3_)_2_ (a,b) in the absence and (c,d) in the presence of gum arabic. The morphology changes in the presence of gum arabic. The star-shaped structures (Figure 11b) became raspberry-like structures of self-assembled aggregates of nanoparticles (Figure 11d) [116].

Table 2 highlights that natural gum-mediated ZnO nanoparticle synthesis is governed by a delicate interplay between chemical supersaturation, polymer–metal coordination, and thermal transformation processes [44]. Among all parameters, precursor concentration and pH emerge as the primary kinetic drivers, as they directly control Zn^2+^ hydrolysis and the formation rate of the Zn(OH)_2_ nuclei. Elevated precursor concentrations or strongly alkaline conditions accelerate nucleation, often yielding smaller primary crystallites but increasing the risk of aggregation if insufficient stabilization is provided. In contrast, gum concentration, molecular weight, and gum-to-metal ratio function as dominant stabilization parameters, modulating ion diffusion, steric hindrance, particle confinement, and surface passivation. Higher gum content generally restricts crystal growth and enhances colloidal stability, although excessive viscosity may impede uniform mixing and broaden size distribution. The chemical structure and molecular weight of the selected gum significantly influence nanoparticle formation. Gums with higher carboxylate content and branching density typically provide stronger metal ion coordination and better stabilization, resulting in smaller particle sizes. Increasing gum concentration enhances capping efficiency and viscosity, leading to reduced particle growth and narrower size distributions. However, excessive gum content may hinder nucleation and result in incomplete crystallization. The pH plays a decisive role in ZnO nanoparticle synthesis by controlling zinc ion hydrolysis and the ionization state of gum functional groups. Alkaline conditions generally favor ZnO formation, while acidic environments stabilize zinc–gum complexes and delay nucleation. Optimal pH ranges promote uniform nucleation and controlled growth, whereas extreme pH values can lead to aggregation or irregular morphologies.

The choice of zinc salt affects solubility, ion release rate, and interaction with gums. Zinc acetate and zinc nitrate are commonly used due to their high solubility and compatibility with aqueous systems. Higher precursor concentrations increase nucleation density but may also promote aggregation if not adequately stabilized by the gum matrix. The most used sources of “Zn^2+^” zinc cation for ZnO synthesis, due to price and availability, are Zn(NO_3_)_2_·6H_2_O, Zn(CH_3_COO)_2_·2H_2_O, ZnCl_2_, ZnSO_4_·7H_2_O, and ZnO respectively, in order of popularity [111,112].

Thermal parameters, particularly reaction temperature and calcination conditions, primarily influence crystallinity and defect density rather than initial nucleation. Reaction temperature influences nucleation kinetics, crystallinity, and defect density. Mild heating accelerates hydrolysis and dehydration processes, enhancing crystallinity without excessive energy input. Increased temperature promotes crystal growth and phase purity but may induce particle coarsening and partial degradation of surface-bound polysaccharides. Similarly, calcination improves crystallinity and removes organic residues, yet it often reduces surface area and alters surface functionality—factors critical for photocatalytic and antimicrobial performance.

Longer reaction times allow for particle growth and shape evolution, while shorter durations favor smaller nanoparticles. Balancing temperature and time is crucial to achieving high-quality ZnO nanostructures.

Process-related variables such as stirring speed, ultrasonication, solvent composition, and drying method, though sometimes underreported, significantly affect dispersion quality and agglomeration behavior. These parameters influence mass transfer and interparticle interactions, thereby indirectly shaping final physicochemical properties. Variations in synthesis parameters directly affect optical, antimicrobial, and photocatalytic properties of ZnO NPs. Smaller particles with higher surface defect densities often exhibit enhanced photocatalytic and antimicrobial activity. Gum-mediated surface functionalization can further influence charge transfer processes and interaction with target species. Atmospheric control during calcination influences defect density, which directly affects photocatalytic performance. Table 3 underscores that successful gum-mediated ZnO synthesis requires coordinated optimization of kinetic, stabilization, and thermal parameters. A balanced approach—ensuring controlled supersaturation, adequate polymer stabilization, and moderated thermal treatment—is essential to achieve nanoparticles with desirable size uniformity, crystallinity, and functional performance for environmental applications. In summary, systematic control of reaction parameters enables tailoring of ZnO nanoparticle properties for specific applications, highlighting the versatility of natural gum-mediated green synthesis.

## 7. Comparative Assessment with Other Green Synthesis Routes

The rapid expansion of green nanotechnology has resulted in the development of multiple biologically inspired strategies for synthesizing ZnO NPs, which include plant extract-mediated synthesis [50,51,52,53,54,55,56,57,58], microbial synthesis (bacteria, fungi, and algae [59,60,61,64]), protein-[62,63], and enzyme-assisted methods [119], and biopolymer-directed routes, such as those mediated by natural polysaccharide gums [11,12,14,18,28,44,77,78,83,84,86,87,88,90,91,96,97,102,103,104,112,113,114,115,116,117,118]. While all these strategies aim to reduce chemical toxicity and energy consumption, they differ significantly in mechanistic pathways, reproducibility, scalability, and application suitability. A critical comparative assessment is therefore necessary to contextualize natural gum-mediated synthesis within the broader landscape of sustainable ZnO nanoparticle fabrication.

Plant extract-mediated synthesis is among the most widely reported green approaches for ZnO nanoparticle production [50,51,52,53,54,55,56,57,58]. Extracts derived from leaves, fruits, peels, roots, and flowers contain diverse phytochemicals such as polyphenols, flavonoids, alkaloids, terpenoids, and proteins. These compounds (amides, carboxylic acids, aldehydes, ketones, sugars, terpenoids, and flavones) function primarily as reducing and capping agents due to the presence of functional groups, facilitating the hydrolysis of zinc precursors and stabilizing the resulting nanoparticles. The principal advantage of plant extract-based synthesis lies in its simplicity and strong reducing capability, often enabling rapid nucleation under mild conditions. Additionally, phytochemical residues on the nanoparticle surface may impart enhanced antimicrobial or antioxidant properties, which can be beneficial for biomedical and environmental applications.

However, both plant extract and gum systems suffer from intrinsic heterogeneity. The concentration and composition of phytochemicals vary with plant species, geographic origin, seasonal changes, and extraction methodology. Such variability can significantly influence nucleation kinetics, particle size distribution, and surface chemistry, leading to limited reproducibility. Furthermore, mechanistic understanding is often hindered by the complex mixture of bioactive molecules present in extracts.

Microbial routes employ bacteria, fungi, or algae to synthesize ZnO NPs through intracellular or extracellular processes [1,59,60,61,64]. In these systems, enzymatic activity, metabolic byproducts, and cell wall components contribute to metal ion reduction and nanoparticle formation. Microbial synthesis offers notable advantages, including operation under ambient conditions and high biocompatibility. Despite these benefits, microbial synthesis presents practical limitations. Cultivation requires sterile conditions, precise nutrient control, and extended incubation periods. Downstream processing—particularly nanoparticle recovery and purification—can be labor-intensive and time-consuming. Moreover, scalability remains challenging due to biological variability and contamination risks. In contrast, natural gum-mediated synthesis avoids living systems, reducing biosafety concerns and simplifying scale-up procedures. Gums are commercially available, inexpensive, and stable during storage, making them more attractive for industrial translation.

Proteins and enzymes have also been explored as templating and reducing agents in ZnO nanoparticle synthesis [62,63,119]. Amino acid residues with carboxyl, amine, and thiol groups interact with zinc ions, enabling nucleation and growth control. These biomolecules offer high specificity and can produce well-defined nanostructures. However, protein-based systems are often costly and sensitive to pH, temperature, and denaturation. Enzymatic activity may be compromised during synthesis, limiting reproducibility. Additionally, purification of protein-coated nanoparticles may require extensive processing. Compared with proteins, natural gums are more chemically stable, less expensive, and less susceptible to environmental fluctuations, providing a more robust alternative for routine nanoparticle fabrication.

Various biopolymers, including chitosan, cellulose derivatives, and starch, have also been employed to synthesize or immobilize ZnO NPs [120,121,122]. These materials share several similarities with natural gums, particularly in terms of functional group availability and stabilization mechanisms. For example, chitosan provides amine functionality for metal binding. While these biopolymers are effective stabilizers, some require chemical modification or crosslinking to achieve desired properties. Chitosan, for instance, exhibits limited solubility under neutral conditions, often necessitating acidic media [123]. In contrast, many natural gums dissolve readily in water without chemical treatment, enabling straightforward, aqueous-phase synthesis. Moreover, certain gums combine high molecular weight with branched architectures that enhance steric stabilization without additional processing steps.

From an environmental perspective, green synthesis routes are generally characterized by reduced reliance on hazardous reagents and lower energy consumption compared to conventional chemical methods. Among green approaches, natural gum-mediated synthesis often operates under mild heating (typically below 100 °C) and aqueous conditions, minimizing solvent toxicity and energy demand. Microwave-assisted gum-based methods further reduce reaction time while maintaining low chemical input. Plant extract and microbial methods similarly employ aqueous systems but may involve additional steps such as extraction, filtration, cultivation, or purification, which increase process complexity and resource use. Life-cycle assessments comparing these methods remain limited; however, the simplicity and commercial availability of natural gums suggest favorable prospects for sustainable scale-up.

Reproducibility is a critical parameter for industrial and regulatory acceptance. Natural gums, being commercially standardized materials with defined compositional ranges, offer superior consistency compared to plant extracts and microbial systems. Their long shelf life and batch uniformity support controlled nanoparticle synthesis with reduced variability.

Scalability also favors gum-mediated approaches. The absence of living organisms eliminates contamination risks and simplifies reactor design. Furthermore, gums are widely used in food and pharmaceutical industries, ensuring established supply chains and regulatory familiarity. These factors collectively position natural gum-mediated synthesis as a promising candidate for translational nanotechnology applications.

In summary, while multiple green synthesis strategies for ZnO NPs have demonstrated environmental and functional advantages over conventional chemical routes, natural gum-mediated synthesis offers a balanced combination of sustainability, mechanistic clarity, reproducibility, and scalability. Plant extracts provide strong reducing capacity but suffer from compositional variability. Microbial systems emphasize biocompatibility yet present operational complexity. Protein- and enzyme-based methods enable structural precision but are cost-intensive and sensitive to environmental conditions. Natural gums occupy an intermediate and highly practical position within this spectrum, combining renewable sourcing, structural consistency, multifunctional chemical activity, and industrial feasibility. This comparative analysis reinforces the rationale for focusing the present review specifically on natural gum-mediated synthesis as a strategically advantageous and environmentally responsible pathway for ZnO nanoparticle production.

## 8. Environmental Applications and Environmental Safety Considerations

Natural gum-mediated ZnO NPs have been increasingly investigated for environmental remediation owing to their photocatalytic activity, antimicrobial performance, and adsorption capacity, combined with the sustainability advantages of biopolymer-assisted synthesis [124,125]. Beyond the green fabrication process, several experimental studies have reported quantifiable performance metrics in pollutant degradation [28,81,114,126], water disinfection [127], soil remediation, and agricultural enhancement [128]. This section summarizes representative findings and integrates them with environmental safety considerations. Table 3 summarizes experimental performance metrics for gum arabic-mediated ZnO NPs in environmental and agricultural applications. Notably, gum-mediated ZnO systems demonstrate high photocatalytic degradation efficiencies for model dyes, effective bactericidal activity at environmentally relevant concentrations, and promising enhancement of plant growth and nutrient uptake in slow-release fertilizer formulations.

**Table 3:** Performance of gum arabic-mediated ZnO NPs in diverse environmental and agricultural applications.

Application	Natural Gum	Target/System	Performance Metric	Key Experimental Result	Reference
Photocatalytic degradation	Gum arabic	Congo red dye (aqueous)	Dye removal efficiency	99.5% degradation in 30 min at 20 mg/L CR, 4 mg/mL ZnO-NPs	[114]
Photocatalytic degradation	Gum arabic	Methyl green dye	Photocatalytic decolorization	Improved decolorization vs. unmodified ZnO (exact % not in abstract)	[81]
Photocatalytic degradation (sunlight)	Gum arabic (calcined)	Blue direct129	Photocatalytic removal	~95% BD129 removal under sunlight; stable over cycles	[78]
Photocatalytic degradation (sunlight)	Gum arabic (calcined)	Methylene blue dye	Photocatalytic removal	~97% MB removal under sunlight; stable over cycles	[28]
Antimicrobial water treatment	Gum arabic	*S. aureus*, *E. coli*	MIC and biofilm inhibition	MIC: 31.25–62.5 µg/mL; 50% biofilm toxicity > 500 µg/mL	[127]
Antibacterial stabilization	Gum arabic	ZnO nanofluids against *E. coli*, *S. aureus*	Long-term antibacterial stability	Stabilized nanofluids have superior antibacterial activity vs. unstabilized	[18]
Agriculture enhancement/slow-release fertilizer	Gum arabic	*Spinacia oleracea* (spinach)	Growth and nutrient uptake	Higher content of proteins (17–47%), phenols (25–60%), Zn uptake (91–106%); 52% reduced Zn leaching vs. commercial	[128]

### 8.1. Photocatalytic Degradation of Organic Pollutants

ZnO is a wide bandgap semiconductor (≈3.2–3.3 eV) capable of generating electron–hole pairs under UV irradiation, leading to the formation of reactive oxygen species (ROS) such as •OH and O_2_•^−^ radicals. These species drive oxidative degradation of persistent organic contaminants. Several studies have demonstrated that gum-mediated ZnO NPs exhibit competitive photocatalytic efficiencies compared with conventionally synthesized counterparts [28,78,81,104,114,118].

**Figure 12:**
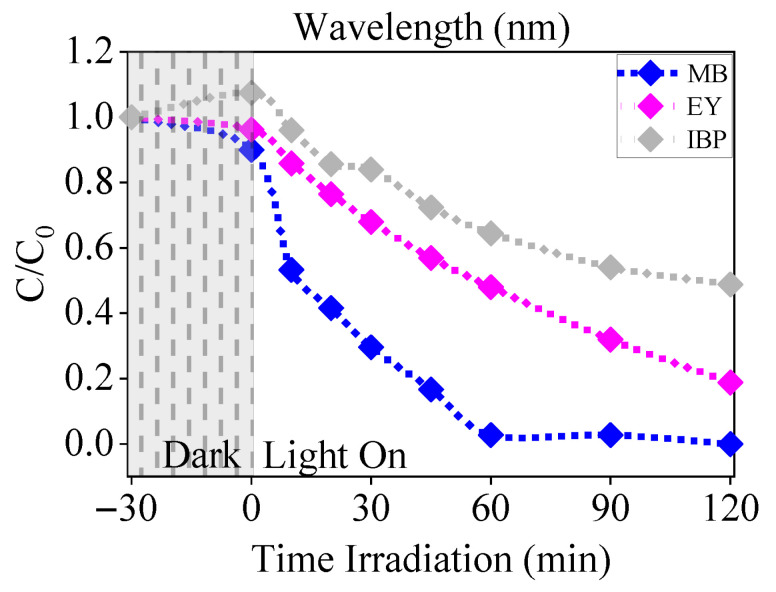
Kinetics of photodegradation for methylene blue (MB), eosin yellow (EY), and ibuprofen using Zn_0.97_Er_0.03_O NPs synthesized using *Mangifera indica* gum as a stabilizing agent (reproduced from [104]).

Photocatalytic experiments were conducted to investigate the effectiveness of Er-doped ZnO NPs synthesized using *Mangifera indica* gum in breaking down methylene blue and eosin yellow dyes, as well as the ibuprofen drug in aqueous solution under UV light exposure. Figure 12 shows the excellent dyes and drug removal efficiency from the solution, which was determined by plotting the concentration ratio C/C_0_ against the irradiation time [104].

Alhasan et al. [114] used as-synthesized ZnO NPs using natural gum arabic as a natural stabilizing agent to photodegrade the toxic Congo red (CR) dye in an aqueous solution, showeing a maximum degradation percentage of 99.5% at pH 8 after 30 min of irradiation. The study demonstrated the suitability of the pseudo-first-order kinetic model for representing the photodegradation process through kinetic studies.

ZnO NPs prepared using gum arabic as capping agent were used to photocatalytically degrade methyl green textile dye. Surface modification with gum arabic leads to change in morphology coupled with reduction in size from 31 to 16 nm. Gum arabic-capped nanoparticles showed efficiency of 95% degrading methyl green, greater than bare ZnO NPs (90%) [81].

Gum arabic ZnO NPs obtained through the sol–gel method demonstrated photocatalytic activity for the removal of as high as 95% of direct blue 129 dye in water under visible light irradiation [78].

ZnO NPs synthesized using gum arabic as a stabilizing agent have shown high photocatalytic degradation efficiency toward methylene blue (MB), achieving >90% degradation within 60–120 min under UV irradiation at catalyst loadings between 0.5 and 1.0 g L^−1^. Enhanced dispersion and reduced aggregation were identified as key contributors to improved activity, attributed to steric stabilization by the polysaccharide matrix [28].

ZnO NPs fabricated using *Abelmoschus esculentus* (okra) mucilage were capable of the complete photodegradation removal of methylene blue (32 mg/L) within 60 min and rhodamine B (9.5 mg/L) within 50 min. The apparent rate constants for the degradation of MB and RhB were determined as 0.0536 min^−1^ and 0.0643 min^−1^, respectively [97].

Cashew tree gum was used as a mediating agent to obtain Ni-doped ZnO NPs through the sol–gel method. Zn_0.99_Ni_0.01_O compound presented the best result in MB degradation (98.4%), reuse tests revealing an efficiency of 98.2% in dye degradation, confirming the stability of the photocatalyst [102].

Guar gum-mediated synthesis of ZnO NPs demonstrated the ability to degrade photocatalytically acrydine orange dye, and a maximum of 98% degradation was obtained after 210 min [129]. Tragacanth gum based hydrogel nanocomposites have been used for the adsorption of methylene blue [130].

ZnO NPs synthesized through a sol–gel route using *Mangifera indica* gum as a macromolecular gelation and templating agent achieved 75% degradation of metronidazole after 120 min, using a catalyst dosage of 0.5 gL^−1^ and a pollutant concentration of 20 mgL^−1^. These results demonstrate that *Mangifera indica* gum enables the eco-friendly synthesis of structurally ordered ZnO with high photocatalytic efficiency and long-term stability for the sustainable removal of pharmaceutical contaminants [118].

Similarly, green-synthesized ZnO NPs have been reported as highly effective, eco-friendly catalysts for the photodegradation of organic dyes efficiently under UV light [124]. Kinetic analyses in such studies typically follow pseudo-first-order behavior, with rate constants enhanced relative to bulk ZnO due to increased surface area and improved adsorption of dye molecules facilitated by residual hydroxyl groups on the nanoparticle surface.

### 8.2. Antimicrobial Water Treatment Applications

ZnO NPs exhibit broad-spectrum antimicrobial activity through ROS generation, membrane disruption, and Zn^2+^ ion release [30]. Gum-mediated ZnO systems have been incorporated into water treatment materials such as coated membranes and polymer composites [4]. When embedded in hydrogel or membrane matrices, gum-mediated ZnO demonstrated sustained antimicrobial performance while limiting nanoparticle leaching [131,132]. One of the most important applications of guar gum nanocomposites is in the field of waste water remediation, thereby playing a significant role in solving the present-day global problem of scarcity of clean water [126]. Guar gum–ZnO nanocomposites used in filtration systems have shown >99% reduction in bacterial load in contaminated water samples within hours of exposure. Importantly, leaching tests revealed controlled Zn^2+^ release, suggesting moderated acute toxicity compared with uncoated ZnO particles. Pectin from unripe banana peel was employed as an efficient ligand in synthesizing ZnO NPs with photocatalytic activity, degrading MB dye (78.5%) after 30.0 min under UV light and bactericidal effect against drug-resistant Gram-positive *Staphylococcus aureus*, but they were ineffective against Gram-negative *Escherichia coli* [88]. *Tamarindus indica* was used as natural reductant in synthesis of ZnO NPs. They exhibited antibacterial activity against the *Klebsiella pneumoniae* MTCC 3384 strain using the agar well diffusion method with inhibition zone of 20.5 ± 0.46 mm at a concentration of 15 mg/mL [96].

ZnO NPs fabricated using *Eucalyptus camaldulensis* gum exhibited inhibitory activity against a pathogenic bacterium (*Escherichia coli*, inhibitory zone 16.6 ± 0.4 mm) and fungal strain (*Schizophyllum commune*, inhibitory zone 18.2 ± 0.9 mm) [84].

ZnO NPs synthesized using gum arabic as a stabilizing agent showed better antibacterial activities against both Gram-positive bacterium *Staphylococcus aureus* (inhibition zone 18.6 ± 0.6 mm) and Gram-negative bacterium *Escherichia coli* (inhibition zone 16.6 ± 0.8 mm), compared to unstabilized ZnO NPs (inhibition zones of 14.0 ± 0.4 mm and 12.0 ± 1.2 mm, respectively) [18].

ZnONPs synthesized using cashew gum and carboxymethylated cashew gum as stabilizing templates presented significant inhibition towards yeasts of the genus *Candida*, particularly *Candida parapsilosis* [103].

Alginate was used for the controlled growth of ZnO NPs, and antimicrobial tests revealed a strong activity against the common pathogens *Staphylococcus aureus* (99.9% reduction) and *Escherichia coli* (100% reduction) after 2 h of exposure [11].

ZnO NPs functionalized with xanthan gum impaired biofilm formation and eradicated preformed biofilms of Gram-negative pathogens, *Chromobacterium violaceum*, and *Serratia marcescens*. ZnO@XG reduced quorum sensing (QS) regulated virulence factors such as violacein (61%) and chitinase (70%) in *C. violaceum* and prodigiosin (71%) and protease (72%) in *S. marcescens* at 128 µg/mL concentration [133].

Guar gum–ZnO NPs significantly enhance the antibacterial activity of ZnO for a range of Gram-negative and Gram-positive bacterial strains and this enhancement was most pronounced for *Bacillus subtilis* and *Salmonella typhi* [134]. Sodium alginate–ZnO NPst exhibited very good antibacterial performance against the Bacillus subtilis (*B. subtilis*), Cellulomonas *cellulans* (*C. cellulans*), *Staphylococcus typhi* (*S. typhi*), and *Escherichia coli* (*E. coli*) bacterial strains, the inhibition zones for *B. subtilis*, *C. cellulans*, *S. typhi*, and *E. coli* being 22, 18, 19.5, and 18.5 mm respectively. Under identical conditions, pure ZnO showed significantly smaller inhibition zones for the corresponding bacterial strains (14, 12.5, 12, and 13.5 mm respectively), while native sodium alginate did not exhibit antibacterial activity [135].

These findings indicate that natural gums not only stabilize ZnO NPs but also enhance compatibility with polymeric matrices, improving their suitability for water purification technologies. Stabilized ZnO nanofluids also persist longer and show better antibacterial performance than unstabilized equivalents.

### 8.3. Heavy Metal Adsorption and Hybrid Remediation

In addition to photocatalysis, gum-mediated ZnO NPs have demonstrated adsorption capability for toxic heavy metals such as Pb^2+^, Cd^2+^, and Cr(VI). ZnO NPs were synthesized using *κ*-carrageenan from *Kappaphycus striatus* as a capping agent and as a source of nonmetal dopants, carbon, and sulfur. *κ*-carrageenan incorporation significantly reduced the band gaps and crystallite sizes of ZnO NPs and demonstrated superior photocatalytic efficiency in degrading methylene blue (99.35%), methyl orange (84.94%), and hexavalent chromium (Cr(VI)) (89.09%) within 120 min of irradiation under UV light, outperforming unmodified ZnO NPs by 1.43, 1.74, and 1.25 times, respectively [86]. Carrageenan-modified ZnO NPs also showed excellent photostability and recyclability, highlighting their potential for eco-friendly water treatment applications.

The adsorption mechanism typically involves electrostatic attraction, surface complexation, and ion exchange. For example, tragacanth gum-assisted nanoparticles have been reported to remove Pb^2+^ from aqueous solutions with adsorption capacities exceeding 50–100 mg g^−1^ under optimized pH conditions (pH 5–6) [130]. Isotherm modeling frequently follows Langmuir behavior, indicating monolayer adsorption. Similarly, guar gum–ZnO nanocomposites have shown significant removal efficiency for hexavalent chromium, with removal percentages above 80% at moderate adsorbent dosages [136]. The presence of surface carboxyl groups enhances metal ion binding affinity, particularly under slightly acidic conditions. Hybrid materials integrating gum-mediated ZnO into biochar or polymeric scaffolds have demonstrated synergistic remediation effects, combining adsorption with photocatalytic reduction (e.g., Cr(VI) to Cr(III)), thereby improving overall detoxification efficiency.

These results indicate that gum–ZnO composites are promising candidates for water purification due to their high efficiency and relatively low, moderate dosage requirements.

### 8.4. Agricultural Enhancement and Soil Applications

**Figure 13:**
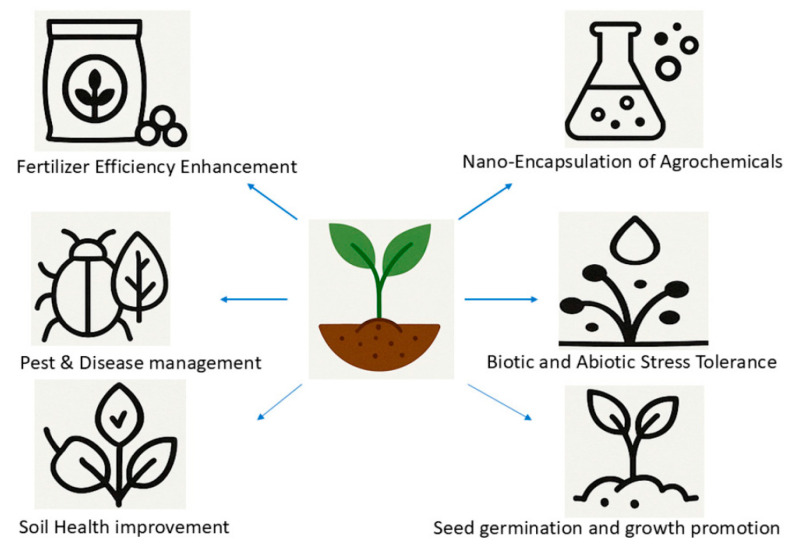
Agricultural applications of ZnO NPs: enhancing crop growth, pest management, and soil health (reproduced from [4]).

Zn is essential for the growth and reproduction of plants, and its deficiency results in losses of crop productivity [137]. Approximately 50% of agricultural soils worldwide are zinc-deficient, leading to poor crop yields and, consequently, low dietary zinc intake in human populations. Moreover, zinc-rich foods are often unaffordable in low-income regions, where deficiency is most prevalent. Zinc deficiency is a major cause of morbidity and mortality, especially in children, pregnant women, and the elderly, as it weakens the immune system, increases infection risks (e.g., diarrhea), and stunts development [137,138]. Pullagurala et al. [139] reviewed conflicting findings regarding the beneficial or detrimental effects of ZnO NPs exposure towards terrestrial biota and indicated that, at low concentrations (about 50 mg/kg), ZnO NPs have beneficial effects on plants, as illustrated in Figure 13. Conversely, at concentrations above 500 mg/kg, they may have detrimental effects, unless there is a deficiency of Zn in the growing medium.

More recently, emerging research proves that green-synthesized ZnO NPs may contribute to agricultural productivity by acting as micronutrient fertilizers and plant growth promoters [35,140,141,142,143,144]. Zinc is an essential micronutrient involved in enzyme activation and protein synthesis. Studies involving biopolymer-mediated ZnO NPs applied to crops such as wheat, maize, and mung bean have reported improved seed germination rates, root elongation, chlorophyll content, and biomass accumulation at low nanoparticle concentrations (typically 10–50 mg L^−1^) [141,145,146]. Compared with bulk ZnO, nanoscale formulations demonstrated enhanced bioavailability and nutrient uptake efficiency. However, concentration-dependent phytotoxic effects have also been observed at higher doses, underscoring the importance of dosage optimization [147,148,149,150]. Surface coating by natural gums may moderate Zn^2+^ release, potentially reducing phytotoxicity compared with uncoated nanoparticles.

ZnO NPs have been observed to increase the growth and yield of numerous food crops. Their administration in the early sowing stages, i.e., seed priming, proved to be effective in improving germination rate, seedling and plant growth and in ameliorating the indicators of plants’ well-being, through several mechanisms such as enhanced nutrients uptake, improved antioxidant properties, ROS accumulation, and lipid peroxidation [151]. In peanuts (*Arachis hypogaea*), ZnO NPs used as a fertilizer induced considerable stimulation of seed germination, seedling vigor, and stem and root growth [148]. An optimum concentration of 1000 ppm of ZnO NPs led to an improvement of dry weight and pod yield of the treated peanuts. Apart from providing the plants with nutrients, ZnO NPs also revive the soil to an organic state without the harmful impacts resulting from the use of a chemical fertilizer, manifested by early flowering and higher leaf chlorophyll content [148]. What gives a nanofertilizer a cutting edge is that it can be used in very low quantities. The inhibitory effect observed with higher nanoparticle concentration (2000 ppm) was ascribed to the accumulation and uptake of ZnO NPs by the roots and reveals the need for judicious usage of these particles in such applications. The key role of the concentration was also reported [145,149]. Stimulated growth was observed in the roots and shoots of mung (*Vigna radiata*) and gram (*Cicer arietinum*) seedlings at an optimum ZnO concentration of 1 ppm and a retarded growth beyond 20 ppm. A ZnO NPs concentration of 1.5 ppm improved shoot dry weight and antioxidant activity in chickpea (*Cicer arietinum*) seedlings [152]. Burman Also, Raliya et al. [153] reported a boost in plant growth and nutrient content in cluster bean (*Cyamopsis tetragonoloba*) seedlings upon treatment with 10 ppm ZnO NPs and enhanced plant height and dry weight were observed in maize plants (*Zea mays*) [146].

ZnO NPs can either promote or inhibit plant development when absorbed by plants and integrated into their transport systems, based on variables such nanoparticle size, concentration, and exposure time. Size is one of the key elements influencing how plants react to ZnO NPs. For example, ZnO NPs with a size of approximately 25 nm have been observed to affect plant growth parameters including root development, blooming, seed germination, seedling vigor index, chlorophyll content, and pod yield [145,149]. Smaller nanoparticles generally exhibit higher surface reactivity and better uptake by plants, which can lead to more pronounced effects on growth and development.

Crop germination rate studies using gum-mediated ZnO NPs are rare. Sharma et al. [128] observed that ZnO–gum acacia nanocomposite significantly improves spinach growth metrics, nutrient content (protein content increased by 17–47%; phenols increased by 25–60%; proline increased by 82–94%; total soluble sugar increased by 20–31%), as well as increases Zn uptake by 91–106% vs. conventional ZnSO_4_ and reduces Zn leaching from soil by ~52% compared to conventional commercial Zn fertilizer, implying strong potential as a slow-release, eco-friendly micronutrient source. It demonstrates that a gum-coated ZnO NP nanocomposite can act as an effective, eco-friendly slow-release Zn fertilizer, enhancing crop performance and nutrient uptake while reducing micronutrient leaching—a major challenge in conventional fertilizer use.

Clusterbean (*Cyamopsis tetragonoloba*) or guar is a vital drought-tolerant legume primarily grown in India for its seeds, which contain 30–33% galactomannan in their endosperm. It is crucial for agriculture as a resilient cash crop, a source of high-protein livestock feed, a nitrogen-fixing soil improver, and a major, high-value agricultural product. Green biosynthesis ZnO NPs foliar sprayed at 10 ppm concentration on 14-day-old clusterbean plants increased plant biomass (27.1%), shoot length (31.5%), root length (66.3%), root area (73.5%), chlorophyll content (276.2%), total soluble leaf protein (27.1%), rhizospheric microbial population (11–14%), acid phosphatase (73.5%), alkaline phosphatase (48.7%), and phytase (72.4%) activity in clusterbean rhizosphere over control in 6-week-old plants due to application of nano-ZnO. The gum content in clusterbean seeds improved by 7.5% after maturity which indicates ZnO in nano form may contribute in agricultural applications [153].

The positive effects of ZnO-NPs on plant growth are thought to result from their ability to improve the availability of essential nutrients, stimulate defense systems, increasing the resistance to infections and help environmental stresses, and enhance photosynthesis, which in turn leads to increased productivity and improved plant performance. To prevent any unfavorable consequences, ZnO NPs application in agriculture must be well regulated. ZnO NPs toxicity can result from overexposure or high concentrations, which can negatively impact plant growth. Therefore, in order to maximize benefits and minimize risks, it is imperative to optimize the dosage and application methods. While these findings are promising and suggest meaningful potential, they have so far been evaluated primarily in controlled settings. As such, their effectiveness and reliability in real-world conditions remain uncertain. These early results should therefore be interpreted with caution, and further large-scale, long-term validation is needed before considering practical use.

### 8.5. Environmental Safety Considerations

Although gum-mediated ZnO NPs are synthesized via eco-friendly methods, their environmental deployment requires careful evaluation [154,155]. Toxicity toward aquatic organisms is strongly influenced by dissolved Zn^2+^ concentration [156]. Surface coatings may reduce dissolution rates, but environmental conditions such as pH and natural organic matter can modify behavior. Available ecotoxicological data for ZnO NPs indicate oxidative stress responses in algae and invertebrates at elevated concentrations. Despite several beneficial roles of ZnO NPs in agriculture, the accumulation of ZnO NPs leads to negative impact on flora and fauna. The process of uptake, translocation and accumulation of ZnO NPs in crops is not studied adequately as many of the studies have commonly been performed until germination stage only. Chronic exposure studies remain limited for gum-mediated systems specifically. Therefore, safe-by-design strategies—such as immobilization in matrices, dose optimization, and recovery after treatment—are critical for minimizing unintended ecological impacts. NPs synthesized by biological entities are more stable and biocompatible and show reduced hazardous impacts [5].

Collectively, the literature evidence demonstrates that natural gum-mediated ZnO NPs exhibit strong performance in photocatalytic degradation [28,78,81,114,126], heavy metal adsorption [86,130,136], water disinfection [127], drug delivery [157], soil remediation, and agricultural enhancement [128,158]. Their improved dispersion, surface functionality, and compatibility with aqueous systems contribute to effective remediation outcomes. However, concentration-dependent toxicity and long-term environmental behavior must be systematically assessed to ensure sustainable implementation. Integrating high remediation efficiency with comprehensive safety evaluation will be essential for advancing gum-mediated ZnO NPs as viable environmental nanotechnologies.

## 9. Challenges, Limitations, and Knowledge Gaps

Despite the growing body of literature supporting natural gum-mediated synthesis of ZnO NPs as a sustainable alternative to conventional methods that aligns strongly with green chemistry principles, several scientific, technological, and regulatory challenges remain in terms of mechanistic clarity, standardization, scalability, and long-term safety evaluation. These limitations must be addressed for advancing this approach from laboratory-scale demonstrations to industrial and environmental applications.

### 9.1. Variability in Gum Composition and Morpho-Structural Complexity

The performance of natural gums in ZnO NPs synthesis is governed by a multifactorial interplay between coordination chemistry, steric effects, electrostatic interactions, and solution rheology. Gums that combine high functional group density (especially –COO^−^), moderate molecular weight, and branching and balanced viscosity tend to provide the most effective control over nucleation, growth, and stabilization. However, due to the inherent variability in natural gum composition, these mechanisms may not operate identically across studies, reinforcing the need for standardized characterization and reporting in future research. Natural gums are not entirely uniform materials, even though they are generally more compositionally defined than plant extracts, their chemical composition can vary depending on botanical origin, harvesting conditions, purification processes, and storage. Variations in molecular weight distribution, branching architecture, and uronic acid content can influence zinc ion complexation, nucleation kinetics, and nanoparticle growth pathways. Standardized gum characterization—covering molecular weight distribution, functional group density, and rheological properties—are not routinely integrated into nanoparticle synthesis studies. This lack of standardized reporting complicates cross-study comparison, limits reproducibility and hampers mechanistic interpretation. The standardization of gum characterization protocols is therefore needed to establish structure–function relationships and enable reliable comparison across studies.

Many published studies report synthesis using broadly defined materials such as “gum arabic” or “guar gum” without detailed characterization (e.g., molecular weight, compositional profile, or impurity content). This lack of standardization complicates efforts to establish clear structure–property relationships and undermines claims of reproducibility. In contrast to synthetic stabilizers with well-defined molecular structures, natural gums introduce an additional layer of variability that is often underreported. Therefore, while natural gums offer clear advantages in terms of sustainability and biocompatibility, claims regarding reproducibility, scalability, and process control must be treated with caution. Future studies should prioritize: compositional characterization of the gum (e.g., FTIR, NMR, carbohydrate profiling), reporting of source and purification method, and implementation of standardized protocols or purified fractions where possible.

Gum-mediated synthesis often produces well-dispersed nanoparticles, but precise control over morphology (e.g., rods, plates, hierarchical structures) remains challenging. Reaction parameters such as pH, temperature, precursor concentration, and gum concentration strongly influence nucleation and growth, yet systematic optimization studies are relatively scarce. Additionally, many reports rely on calcination steps to convert intermediate zinc hydroxide phases to ZnO. High-temperature treatment may partially degrade organic coatings, altering surface chemistry and potentially negating some advantages of biogenic stabilization. Developing low-temperature or template-preserving strategies represents an important research direction.

Addressing this variability is essential for transitioning gum-mediated ZnO nanoparticle synthesis from laboratory-scale demonstrations to reliable and industrially scalable processes, and for ensuring that reported performance enhancements are genuinely attributable to controlled material properties rather than uncontrolled natural variability.

### 9.2. Limited Mechanistic Elucidation

While it is widely accepted that polysaccharide functional groups such as hydroxyl (–OH) and carboxyl (–COOH) moieties are key contributors to Zn^2+^ ions chelation and nanoparticle stabilization, detailed mechanistic understanding at molecular-level remains insufficient. Metal ion complexation, hydrolysis kinetics, nucleation rate, and polymer network confinement are often inferred rather than directly demonstrated.

Advanced spectroscopic techniques (e.g., in situ time-resolved FTIR, X-ray absorption spectroscopy) and computational modeling remain underutilized in this field. Consequently, precise structure–function correlations between gum composition (e.g., branching degree, molecular weight, uronic acid content) and nanoparticle characteristics are not yet fully established. Without deeper mechanistic insight, rational design of gum-mediated systems for targeted particle sizes or morphologies remains largely empirical.

### 9.3. Scalability and Process Integration

While natural gum-mediated synthesis of ZnO nanoparticles offers clear advantages in sustainability and functionality, translating this approach from laboratory research to real-world applications requires addressing several critical challenges. From an industrial perspective, scalability remains insufficiently demonstrated. Most studies are conducted at laboratory scale with small reaction volumes. Even though gum-mediated synthesis is a priori conceptually scalable, due to aqueous processing and commercial availability of biopolymers, few studies have demonstrated pilot-scale production. One of the most pressing challenges is the scaling of gum-mediated ZnO NPs synthesis from bench-scale to industrial production. Laboratory protocols typically rely on batch processes with controlled stirring, temperature, and reagent addition, which may not be directly transferable to large-scale systems. Transitioning to pilot-scale production requires addressing issues such as mixing efficiency, viscosity management, reaction time optimization, and product purification. High viscosity systems (common with gums) can hinder uniform mixing, leading to non-uniform nucleation and broad particle size distributions. The influence of subtle variations in gum purity and source on nanoparticle formation is insufficiently documented. Even commercially available gums may contain minor protein fractions or mineral residues that influence reaction pathways. A systematic comparison of well-characterized gum fractions is needed to decouple the effects of polymer architecture from extraneous components. Furthermore, the integration of gum-mediated synthesis into continuous-flow or industrial reactor systems has not been extensively explored. Efficient separation, washing, and drying of nanoparticles must be developed to avoid aggregation and preserve surface functionality. While gums are relatively inexpensive, their price and availability may fluctuate depending on agricultural production and regional supply, process optimization, purification, and quality control may influence overall economic feasibility; however, economic feasibility analyses, including cost comparisons with conventional precipitation or sol–gel methods, are largely absent from the literature. Future work should focus on process engineering strategies, including reactor design and rheology control, to ensure consistent and economically viable large-scale production.

### 9.4. Environmental and Toxicological Data Gaps—Regulatory Challenges

The environmental behavior of gum-coated ZnO NPs remains incompletely characterized. Although green synthesis reduces chemical hazards during fabrication, comprehensive long-term ecotoxicological assessments are limited. Most studies focus on short-term antimicrobial or cytotoxicity assays rather than chronic ecotoxicological effects, bioaccumulation, or trophic transfer effects. Additionally, the environmental transformation of gum-coated ZnO NPs—such as dissolution, aggregation, or surface modification under natural conditions—remains insufficiently characterized, as well as the degradation pathways of the polysaccharide coating.

For practical deployment, ZnO NPs must be integrated into functional systems, rather than used as dispersed powders: immobilized in membranes and coatings for water treatment, reducing nanoparticle release and enabling reuse; incorporated into polymer composites or hybrid systems combining ZnO with biochar, clays, hydrogels or other supports to enhance adsorption and photocatalysis for controlled pollutant removal or encapsulated in soil matrices for slow-release agricultural applications. Integration strategies must balance performance, stability, and environmental safety, ensuring that nanoparticles remain effective while minimizing unintended release into ecosystems.

Comprehensive life-cycle assessment (LCA) studies specific to gum-mediated ZnO systems are particularly scarce. Without quantitative sustainability metrics, claims of environmental superiority remain largely qualitative, accurate risk assessment, and regulatory evaluation remain incomplete.

In summary, while natural gum-mediated synthesis of ZnO NPs represents a promising and environmentally aligned approach, significant challenges persist in mechanistic understanding, process optimization, scalability, and comprehensive risk assessment. To overcome these limitations, future research should prioritize mechanistic elucidation using advanced analytical tools, establish standardized synthesis and reporting guidelines, conduct life-cycle and techno-economic assessments, and expand comprehensive toxicological studies. Addressing these knowledge gaps will be critical to transitioning natural gum-mediated ZnO NPs synthesis from promising laboratory research to reliable, industrially viable green nanotechnology.

## 10. Conclusions and Future Research Directions

The eco-friendly synthesis of ZnO NPs mediated by natural gums represents a compelling convergence of green chemistry principles, renewable biomaterials, and functional nanotechnology. As highlighted throughout this review, natural gums—owing to their polysaccharide-rich composition, abundant hydroxyl and carboxyl functional groups, biodegradability, and commercial availability—serve as multifunctional mediators capable of coordinating zinc ions, directing nucleation, and stabilizing nanoparticle growth within aqueous systems. Compared with conventional chemical routes and other biogenic approaches, gum-mediated synthesis offers a favorable balance between sustainability, reproducibility, and process simplicity.

The distinctive advantage of natural gums lies in their dual functionality as both templating and stabilizing agents. Their macromolecular architecture enables steric stabilization and controlled particle growth without requiring synthetic surfactants or hazardous reducing agents. Moreover, their compatibility with aqueous processing conditions and moderate reaction temperatures aligns well with energy-efficient manufacturing strategies. These characteristics position gum-mediated synthesis as a viable candidate for sustainable production of ZnO NPs for environmental, antimicrobial, catalytic, and potentially biomedical applications. Nevertheless, to fully realize the potential of this approach, several strategic research directions must be prioritized:

- First, deeper mechanistic understanding is essential. Establishing clear structure–function relationships between gum composition, molecular weight, branching degree, and nanoparticle properties will enable predictive design rather than trial-and-error optimization.
- Second, systematic parameter optimization studies are needed to achieve tighter control over particle size distribution, morphology, and crystallinity.
- Third, scalability and techno-economic evaluation must be addressed to clarify their commercial competitiveness.
- Fourth, robust environmental and toxicological life-cycle assessment studies are indispensable for the validation of sustainability advantages and regulatory acceptance.
- Fifth, interdisciplinary integration offers promising avenues for innovation. Hybrid materials combining gum-mediated ZnO NPs with biodegradable polymers, biochar, or membrane systems could yield multifunctional platforms for water purification and pollutant remediation. Furthermore, functionalization strategies leveraging gum chemistry may enable targeted surface modification for enhanced selectivity or catalytic efficiency.

In conclusion, mechanistic research, process optimization, environmental evaluation, and cross-disciplinary collaboration will be instrumental in advancing to scalable, environmentally responsible applications. By aligning material performance with principles of renewable sourcing, low toxicity, and energy efficiency, gum-mediated ZnO nanoparticle synthesis has the potential to contribute to the next generation of circular economy-oriented technological solutions.

## Figures and Tables

**Table 1:** Natural gums reported as mediators in green synthesis of ZnO NPs.

Natural Gum	Source	Key Chemical Constituents/Functional Groups	Role in ZnO NP Synthesis	Reference
Gum arabic	Plant exudate (*Acacia* spp.)	Arabinogalactan polysaccharides, glycoproteins –OH, –COOH, –NH	Reducing agent, stabilizer, biotemplate, and size control	[14,18,19,28,77,78,79,80,81]
Guar gum	Seed gum (*Cyamopsis tetragonoloba*)	Galactomannan –OH	Chelating and steric stabilization	[19,82]
Xanthan gum	Microbial (*Xanthomonas campestris*)	β-D-glucose –OH, –COO^−^	Stabilization and dispersion of ZnO NPs	[19,83]
Eucalyptus camaldulensis gum	Plant exudate	Mixed acidic polysaccharides –OH, –COOH	Biogenic matrix and stabilizer	[84]
Gum karaya	Plant exudate (*Sterculia* spp.)	Galactose, rhamnose, galacturonic acid –OH, –COOH,		[79]
Moringa oleifera gum	Plant exudate	Arabinogalactan Sugars, flavonoids (–OH)	NP syntheses and stabilization	[85]
Alginate	Brown algae	β-D-mannuronic acid (M) and a-L-guluronic acid		[11,12]
Carrageenan	Marine polysaccharide (red algae)	Sulfated galactans –OH, –SO_3_^−^	Electrostatic stabilization and templating	[86]
Pectin	Banana peel	Arabinose, xylose, galacturonic acid, –OH, –COOH,		[87,88,89,90]
Gum tragacanth	Plant exudate (*Astragalus* spp.)	Tragacanthin, bassorin (acidic polysaccharides) Key polysaccharide –OH, –COOH	Metal ion chelation and steric stabilization	[91,92,93,94]
Locust bean gum	Seed gum (*Ceratonia siliqua*)	Galactomannan –OH	Steric stabilization, nucleation control, and biotemplate	[95]
Tamarind gum	Seed polysaccharide (*Tamarindus indica*)	Xyloglucan –OH	Capping and viscosity control	[96]
Okra gum	Plant polysaccharide (*Abelmoschus esculentus*)	Rhamnogalacturonan –OH, –COOH	Stabilizer and dispersion agent	[97,98]
Gum cordia	Plant exudate (*Cordia myxa*)	Galactose, rhamnose, mannose, uronic acids	Reducing and stabilizing agent	[99]
Asafoetida gum	Plant exudate (*Ferula asafoetida*)	Decenals, methoxy-4-vinylphenol, octadecadiynoic acid	Reducing and capping agent	[100]
Gum kondagogu	Plant exudate (*Cochlospermum gossypium*)	Galactose, rhamnose, uronic acids	Reducing and stabilizing agent	[101]
Cashew gum	Seed polysaccharide (*Anacardium occidentale*)	Galactose, arabinose, rhamnose, glucuronic acid	Gel-based templating and stabilization	[102,103]
Mangifera indica gum	Plant exudate	Sesquiterpenes	Stabilizing agent	[104]
Taro gum	Plant exudate (*Colocasia esculenta*)	Arabinose, xylose, mannose, galactose Arabinogalactan	Stabilizing agent	[105]

**Table 2:** Influence of reaction parameters on natural gum-mediated ZnO NPs synthesis.

Parameter	Typical Range	Mechanistic Role	Effect on Nucleation and Growth	Impact on Physicochemical Properties
Zinc precursor type (e.g., acetate, nitrate, sulfate)	0.01–0.5 M	Determines hydrolysis rate and ionic strength	Faster hydrolysis (e.g., nitrate) → rapid nucleation; acetate → controlled growth	Affects crystallinity, defect density, morphology
Precursor concentration	0.01–1.0 M	Controls supersaturation level	High concentration → burst nucleation, possible aggregation	Smaller size at optimal levels; excessive concentration → polydispersity
Gum concentration	0.1–5 wt%	Provides chelation and steric stabilization	Higher concentration → restricted growth	Reduced aggregation, improved dispersion, possible smaller particles
Gum molecular weight	10^3^–10^6^ Da	Influences viscosity and diffusion	High MW → slower ion diffusion, confined nucleation	Enhanced stabilization; possible broader size distribution if overly viscous
Gum-to-metal ratio	1:1–10:1 (mass basis)	Determines coordination density	High ratio → limited crystal growth	Smaller crystallites, improved colloidal stability
pH of reaction medium	7–12	Controls Zn^2+^ hydrolysis and Zn(OH)_2_ formation	Alkaline pH favors rapid nucleation	Influences phase purity, particle size, and surface charge
Type of base (NaOH, KOH, NH_4_OH)	0.1–2.0 M	Regulates hydroxide ion availability	Strong bases → faster nucleation	Alters morphology (rods, spheres), affects crystallinity
Reaction temperature	25–100 °C	Affects kinetic energy and diffusion	Higher temperature → faster growth	Increased crystallinity; possible particle coarsening
Heating method (conventional vs. microwave)	—	Influences nucleation uniformity	Microwave → homogeneous rapid nucleation	Narrower size distribution; enhanced crystallinity
Reaction time	30 min–24 h	Determines growth duration	Longer time → particle growth and ripening	Larger crystallite size; improved crystallinity
Stirring speed	200–1000 rpm	Enhances mass transfer	Improved mixing → uniform nucleation	Reduced agglomeration; improved dispersion
Ultrasonication	5–60 min	Disrupts agglomerates; enhances mixing	Promotes uniform nucleation	Smaller particle size; improved stability
Calcination temperature	300–600 °C	Converts Zn(OH)_2_ to ZnO; removes organics	Higher temperature → grain growth	Increased crystallinity; possible loss of surface functional groups
Calcination duration	1–4 h	Affects phase transformation	Extended duration → sintering	Larger particle size; reduced surface area
Drying method (oven vs. freeze-drying)	—	Influences particle aggregation	Freeze-drying reduces capillary collapse	Higher surface area; reduced aggregation
Solvent system (water vs. water–ethanol)	—	Alters solubility and diffusion	Mixed solvents → modified nucleation kinetics	Changes morphology and particle dispersion
Ionic strength of medium	Variable	Screens electrostatic interactions	High ionic strength → reduced electrostatic repulsion	Increased aggregation tendency
Presence of crosslinkers	Low wt%	Alters gum network density	Enhanced confinement of Zn^2+^	Improved uniformity; controlled growth
Aging time before calcination	1–24 h	Allows structural rearrangement	Promotes Ostwald ripening	Larger crystals; improved structural ordering
Atmosphere during calcination (air, inert)	—	Controls oxidation environment	Inert → defect formation	Influences oxygen vacancies and photocatalytic activity

## Data Availability

No new data were created or analyzed in this study. Data sharing is not applicable to this article.

## Abbreviations

The following abbreviations are used in this manuscript:

FTIR	Fourier transformed infra-red
NPs	nanoparticles
UV	ultraviolet
